# Supplementary material for: Learning curve and surgical outcome of robotic assisted colorectal surgery with ERAS program
Source: Sci Rep. 2022 Nov 29;12:20566. doi: 10.1038/s41598-022-24665-w (PMC9709162; doi:10.1038/s41598-022-24665-w)
Supplement: Supplementary file 1 — Supplementary Information 1. [file 41598_2022_24665_MOESM1_ESM.zip › LSC Raw data-Ñ[▒K/125/H_ñw╝╨░O▒Kñσ.pdf]

正式摘要(電子簽章：000000214781535)		
W52 / W52 ☐英文標題(注意:無英文姓名-請補病人基本檔!)	☐【讀取】憑證	

臺中榮民總醫院
出院病歷摘要

轉入號 : 轉出號 :

[redacted]

(8)流水編號	
(9)入院日期	110/04/13 (2021) 直腸外科 W52 - 152
(10)轉科日期	
(11)出院日期	110/04/19 (2021) 住院天數計 6 日

診斷

- 入院 :S colon tumor
- 出院 :1. S colon tumor 20 cm FAV s/p Laparoscopic anterior resection with end-to-end anastomosis on 2021/4/14

主訴

- colon polyps due to stool OB(+)

病史

- This is a 63-year-old male without past medical history
  According to the patient, he found colon polyps due to stool OB(+). He was therefore admitted for laparoscopic Anterior resection and anastomosis. He is totally ADL independant. He denies any allergy or family history.

體檢發現

- 2021/4 To cecum, A colon diverticum.
  T colon polyp s/p EMR hot snare, 60 cm FAV
  S colon tumor s/p Bx 15 cm FAV. Tatoo.
  - General appearance :
    - easy looking , conscious clear , without cardiopulmonary distress
    - Vital signs自動帶入date 體溫(BT) 脈搏(P) 呼吸(R) 血壓(BP) 身高(BH) 體重(BW) BMI
      20210413 36.4℃ 75次/分 16次/分 121/85mmHg 165.3cm 68.8kg 25.2
    - Vital signs: B.P. 121/85 mmHg , P.R. 75 /min , R.R. 16 /min , B.T. 36.4 ℃
  - HEENT :
    - Eyes: no anemic conjunctiva, no icteric sclera, normal EOM
    - ENT: no otorrhea, no rhinorrhea, no erythematous mucosa, no injected tonsil, no deformity
  - Neck: supple, freely movable, trachea midline,no deformity, no engorged jugular vein , no palpable thyroid or lymph node enlargement
  - Chest:
    - Inspection: Grossly normal, symmetric and fully expansion, no accessory muscle used, no operation scar, no spider angiomata, no gynecomastia
    - Palpation: no palpable mass or nodule, no subcutaneous emphysema, no tenderness
    - Ausculation: clear breathing sound
  - Heart:
    - Percussion: normal heart size
    - Ausculation: regular heart beat, normal S1, S2, no gallop, no murmur
  - Abdomen:
    - Inspection: in
      - flat contour, symetrically, no scar, no stria, no dilated veins, no rashes
    - Ausculation: normoactive bowel sound, no abdominal bruits
    - palpation: soft, no muscle guarding or rigidity, no local tenderness, no

- rebounding pain, no palpable mass, no palpable pulsation
    - Percussion: no shifting dulllness, no hepatomegaly, no splenomagaly
-

Peripheral pulsation :	CA	BA	RA	FA	PA	PTA	DPA
Right side :	++	++	++	++	++	++	++/+++
Left side :	++	++	++	++	++	++	++/+++

- Genito-anus: no tenderness or palpable mass lesion or rectal shelf, normal external genitalia, normal anal tone, brown stool

CRS CONDITION

-
    - Lung

手術日期及方法

- 手術日期及方法20210414 Laparoscopic Anterior resection and anastomosis - malignant

住院治療經過

- The patient was admitted for scheduled surgery with Laparoscopic Anterior resection and anastomosis. During hospitalization, pre-operative survey showed acceptable conditions. He underwent surgery on 2021/4/14. After surgery, he went back to ward for post-operative care. He dinied any discomfort after surgery. Under relatively stable condition, he was therefore discharged with OPD follow-up.

合併症

- nil

一般檢查

- 累積報告 :

DATE	TIME	ALB	TP	BIL.T	BIL.D	ALKP	AST	ALT	LDH	CHOL	TG	HDL-C	LDL	UA	NH3	AMY
1100414	1746	4.1		0.81	0.21		19	16								

DATE	TIME	WBC	RBC	HGB	HCT	MCV	PLT	BLST	NEUT	BAND	SEG	LYM	MON	EOS	BAS
1100414	1743	12180	5.02	14.5			156		91.7			7.1	1.0	0.0	0.2

DATE	TIME	NA	K	CL	CA	BUN	CREAT	Mg	P	CRP	ESR	PCT	LIPASE	LACTATE
1100414	1746	142	3.5	106	8.5	9	0.89							

特殊檢查

病理報告

其它

出院時情況

- 3.改門診治療

出院指示

-

藥名	劑量	劑量單位	用法	頻次	數量	數量單位	現況	天數	單位
Mocolax tab 400mg	1	TAB	PO	TID	21	TAB	TH	7	天
(口)Takepron OD tab 30mg	1	TAB	PO	QDAC	7	TAB	TH	7	天
Ultracet tab	1	TAB	PO	Q8H	9	TAB	TH	3	天

出院計劃

- 門診日期
    - date on 2021/04/26

主治醫師 2222H 林俊余 / 住院醫師 9958C 林強生

正式摘要(**電子簽章：000000214781535)**

W52 / W52

doctorRank:B
